# Life-Course Trajectories of Childless Women: Country-Specific or Universal?

**DOI:** 10.1007/s10680-022-09624-5

**Published:** 2022-06-09

**Authors:** Valentina Tocchioni, Anna Rybińska, Monika Mynarska, Anna Matysiak, Daniele Vignoli

**Affiliations:** 1 Department of Statistics, Computer Science, Applications “G. Parenti”, University of Florence, Florence, Italy; 2 Center for Child and Family Policy, Duke University, Durham, USA; 3 Institute of Psychology, Cardinal Stefan Wyszyński University in Warsaw, Warsaw, Poland; 4 Interdisciplinary Centre for Labour Market and Family Dynamics, Faculty of Economic Sciences, University of Warsaw, Warsaw, Poland

**Keywords:** Childless women, Life-course, Sequence analysis, Discrepancy analysis, Cluster analysis

## Abstract

While existing research has documented complexities in biographies of childless women, few studies to date have systematically examined the life-course pathways of the childless from a comparative, cross-country perspective. In this paper, we analyse biographies of childless women in four countries—Germany, Italy, Poland, and the United States—in order to investigate whether pathways into childlessness are country-specific or commonly shared across institutional, cultural, and geographical settings. Partnership, education, and employment histories are examined using sequence analysis with dynamic Hamming distance and cluster analysis. Discrepancy analysis indicates a country-effect in women’s biographies although life-course patterns identified in each country share similarities. Overall, seven life-course trajectories have been identified, with the most numerous cluster comprising single, working women who completed their education at a relatively young age. The results highlight a marked variation in the life-courses of childless women. Put together, these findings provide descriptive evidence for both country-specificity and cross-country similarity in the pathways to childlessness.

**Supplementary Information:**

The online version contains supplementary material available at 10.1007/s10680-022-09624-5.

## Introduction

Due to a widespread increase in permanent childlessness across low-fertility countries (Brini, 2020; Kreyenfeld & Konietzka, 2017; Rowland, 2007), recent decades have seen considerable research into the process of remaining childless. Although childlessness is a clearly defined state at every point of the life course, the process of family formation and the path to parenthood unfold over the course of one’s life (Keizer et al., 2008; Mynarska et al., 2015). While singleness continues to be an important factor in the process of remaining childless, the role of professional and educational choices is increasingly acknowledged in the literature (e.g. Heaton et al., 1999; Köppen et al., 2017; Tanturri & Mencarini, 2008). Additionally, recent research has highlighted marked heterogeneity in the socio-demographic characteristics of childless individuals (Mynarska et al., 2015; Tocchioni, 2018).

Heterogeneity in facets of childlessness is also visible in comparative, cross-country studies. Despite the fact that the majority of research on childlessness has applied a country-specific perspective (e.g. Chudnovskaya, 2019; Ciritel et al., 2019; Mynarska & Rytel, 2020), a handful of studies show a marked variation in childlessness trends (Sobotka, 2017) and correlate across countries (Beaujouan et al., 2016; Miettinen et al., 2015; Rijken & Merz, 2014).

In this paper, we examine both life-course heterogeneity and cross-country heterogeneity in the process of remaining childless. We adopt an exploratory approach to study a diversity of life-course trajectories related to having no offspring in four countries—Germany, Italy, Poland, and the United States. These countries represent examples of societies characterised by relatively high childlessness rates, but they also differ markedly from one another with regards to economic conditions, the labour market, family structures, and welfare provisions. We focus on women born between the late 1950s and the 1960s, considering three dimensions of their life-courses: partnership histories, employment spells, and educational attainment. We use sequence analysis, together with discrepancy and cluster analysis, to re-construct life trajectories of childless women in these countries, quantify the heterogeneity in the childless life-course between the countries and, finally, classify childless women in each country into sub-populations that follow similar biographic developments.

Our findings reveal a large diversity in life experiences of childless women both within and between countries: the childless universe does not appear to be uniquely formed by highly educated, working women; but also, by women who left the educational system at a relatively young age, or women with a very weak attachment to the labour market. We further show pathways to childlessness that are universal across different contexts, as well as those unique to a specific country. Our approach highlights the variety of paths that can result in a childless life in different institutional settings, which could, in turn, affect future fertility trends and networks of intergenerational support. This is particularly crucial in countries such as Italy and Poland that rely heavily on the presence of children as key actors of care organisation in old age (Tanturri, 2016).

## Childlessness over the Life-Course

Childlessness, defined as an absence of biological, adopted, or foster children (though not an absence of childrearing responsibilities) is increasingly common, as a growing number of people remain childless, due to either individual choices or life contingencies, or a combination of both (McQuillan et al., 2012; Rybińska & Morgan, 2019). As relevant research expanded, events in three life spheres—partnership, education, and employment status—have been identified as major contributors to the process of remaining childless (Koropeckyj-Cox & Call, 2007; Lee & Gramotnev, 2006).

Singlehood and partnerships play a key role in contemporary childlessness (Berrington, 2017; Jalovaara & Fasang, 2017; Rotkirch & Miettinen, 2017). Union histories may affect individuals’ life-courses, and lead to childlessness either because of a lack of a partner or union dissolution (Keizer et al., 2008; Thomson et al., 2012). Additionally, childlessness has recently been linked to the increasing complexity of union histories (Hart, 2018). Even within stable unions, disagreement on parenthood timing or family size as well as fecundity problems could lead to permanent childlessness (Fiori et al., 2017; Letherby, 1999; Tanturri & Mencarini, 2007).

Although the effects may differ depending on institutional settings (Neyer et al., 2017), prolonged education and labour market participation are associated with a higher probability of remaining childless (Abma & Martinez, 2006; Dorbritz, 2008; Hara, 2008; Hayford, 2013; Keizer et al., 2008). The most common explanation relates to the postponement mechanism. As women remain in education longer, and then strategically postpone childbearing so as to secure their career prospects (Gustafsson, 2001), they could well find themselves at an age where female biological fecundity is reduced (Velde & Pearson, 2002). This could, consequently, lead to childlessness. However, this is but one of the many ways in which education and employment can affect childlessness. Research has also shown that the opportunity costs of motherhood are especially high for those with higher educational attainment (Barthold et al., 2012), thereby suggesting that highly educated women with continuous employment careers may deliberately forego childbearing. At the macrolevel, whilst in some countries childlessness is positively associated with educational level (Berrington, 2017; Berrington et al., 2015), in other settings childlessness is prevalent amongst less-educated women (Miettinen et al., 2015). At the same time, the increased prevalence of childlessness is often coupled with parenthood delay (Berrington et al., 2015).

On the other hand, difficulties within the labour market, and prolonged spells of unemployment, may serve to increase women’s economic uncertainty and negatively impact their financial situation, both of which may lead to childbearing postponement and childlessness (Baudin et al., 2015; Busetta et al., 2019; Mynarska et al., 2015; Vignoli et al., 2020). As this effect might be particularly pronounced among women with lower levels of educational attainment (Baudin et al., 2015), it is evident that the role of employment needs to be considered jointly with educational attainment when investigating the causes of childlessness.

The above literature review suggests that partnerships, education, and employment trajectories that unfold over the course of one’s life have a noticeable impact on childlessness. To capture this diversity in life pathways, a new generation of studies have used sequence analysis to jointly analyse different life spheres over time and their connection to permanent childlessness (Jalovaara & Fasang, 2017; Mynarska et al., 2015; Tocchioni, 2018). We seek to detect—with an analogous approach—the regularities and differences in the paths to childlessness along the three life spheres of partnership, education, and employment. Through this analysis, we expand on a previous study of Mynarska et al. (2015), which compared childless women in Italy and Poland, and examine country-specific types of childless women not only in Italy and Poland but also in Germany and the United States, which represent an array of family formation patterns and welfare state settings.

## Country Contexts

**Fig. 1:**
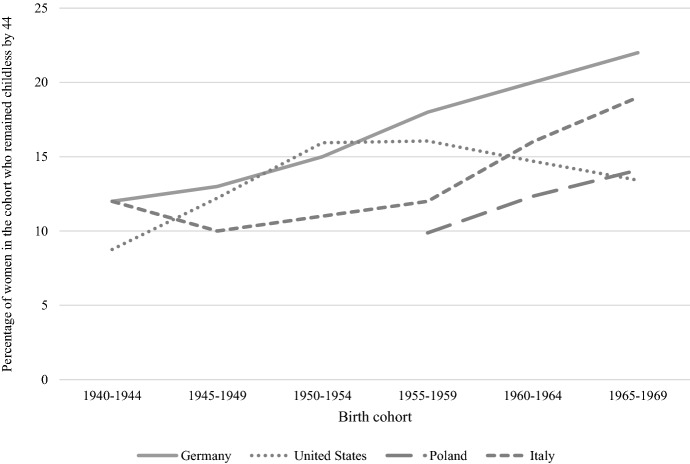
Rates of permanent childlessness by age 44 by birth cohort and country. Germany, Italy, Poland, and the United States. *Source:* Germany: Kreyenfeld & Konietzka, 2017; Italy: Istat (dati.istat.it); Poland and the United States: Human Fertility Database (https://www.humanfertility.org)

The four countries, covered in our study—Germany, Italy, Poland, and the United States—represent distinct geographical regions in the world, namely Continental, Southern, and Central & Eastern Europe, and North America. They differ not only in terms of history, economics, and institutions, but also in family life patterns. In this section, we provide a brief description of similarities and difference between these countries in terms of fertility rates, as well as education, employment, and partnership indicators—measured at approximately the time when the women in the studied cohorts reached young or mid-adulthood (with certain data limitations, see Fig. 1 and Table 1).

**Table 1:** Selected indicators on fertility, partnership, education, and employment in Germany, Italy, Poland, and the United States. *Source*: Unless otherwise stated, OECD database 1990 (https://data.oecd.org/)

	Germany	Italy	Poland	US
Percent of childless women (1965 cohort)	22.0%^a^	18.4%^b^	13.4%^c^	14.0%^c^
Women’s mean age at first child (1990)	26.9^d^	26.9^d^	23.3^c^	24.3^c^
Total Fertility Rate (1990)	1.45	1.36	1.99	2.08
Women’s mean age at first marriage (1990)	25.5^e^	25.9^e^	23.0^f^	25.0^g^
Crude marriage rate (1990)	6.6^e^	5.6^e^	6.7^e^	9.8^g^
Total first marriage rate—women (1990)	0.64^e^	0.70^e^	0.71^f^	0.71^g^
Crude divorce rate (1990)	1.9^e^	0.5^e^	1.1^e^	4.7^h^
Percent of unions that began as cohabitations	66.4%^i^	10.5%^i^	5.8%^i^	54.0%^j^
Divorces per 100 marriages (1990)	30.0^e^	8.7^e^	16.6^e^	42.0^k^
Percent tertiary educated people aged 25–34 (1990)	21.2%^l^	6.6%^l^	14.6%^m^	24.0%
Percent tertiary educated women aged 25–34 (1990)	19.5%^l^	6.4%^l^	18.4%^m^	23.6%
Labour force participation rate people aged 15–64 (1990)	67.4%	59.5%	69.4%^n^	76.5%
Employment rate people aged 15–64 (1990)	66.1%^e^	52.2%^f^	58.8%^o^	72.2%
Employment rate women aged 15–54 (1990)	55.0%^f^	35.9%^f^	52.1%^o^	64.0%
Percent of women working part-time (1990)	39.0%^p^	29.3%^p^	19.7%^p^	38.8%^p^
Percent of NEET people aged 15–29 (1997)	14.3%	25.7%^r^	18.8%	12.6%
Percent of NEET women aged 15–29 (1997)	18.1%	30.5%^r^	24.6%	16.7%

^a^Kreyenfeld & Konietzka, 2017;

^b^Istat (https://www.istat.it/);

^c^Human Fertility Database (https://www.humanfertility.org);

^d^UNECE Statistical database (https://unece.org/data);

^e^Eurostat (https://eurostat.ec.europa.eu), 1990;

^f^Eurostat (https://eurostat.ec.europa.eu), 1993;

^g^Clarke, 1995a;

^h^Clarke, 1995b;

^i^Kalmijn, 2007;

^j^1990–1995, Bumpass & Lu, 2000;

^k^females only, 1995, Schoen & Standish, 2001;

^l^OECD (https://data.oecd.org/), 1991;

^m^OECD  (https://data.oecd.org/), 1995;

^n^OECD  (https://data.oecd.org/), 1992;

^o^Eurostat (https://eurostat.ec.europa.eu), 1997;

^p^International Labor Organization (https://www.ilo.org), Italy and Germany—1990, US—1994, Poland—1997;

^r^OECD, 1998;

Our study covers women born in the late 1950s and 1960s. Steady increases in childlessness were reported for these cohorts in Germany, Italy, and Poland, with the proportion of childless women being the lowest in Poland and the highest in Germany. In the United States, however, the pattern diverges. The increase occurred earlier and, for the analysed cohorts, a downward trend can be observed.

The youngest women in our sample entered adulthood approximately in 1990. In the 1990s, women in Poland and the United States, on average, transitioned to motherhood at a younger age (in their early 20s) and went on to have markedly more children (on average) compared to women in Germany and Italy. While women married on average between 23 and 26 in all four countries, marriage rates were higher in the United States compared to the European countries. Furthermore, while divorce rates were remarkably low in Italy and Poland, they were relatively high in Germany and the United States where, in 1990, out of 100 marriages, respectively, 30 and 42 ended in divorce.

Except for Italy, women’s labour market participation was similar across the countries in the considered period. Among women aged 15–54, the employment rates spanned from 35.9% in Italy to 64.0% in the United States, with Germany and Poland closer to the United States with rates of 55.0% and 52.1%, respectively. Regarding education, the prevalence of tertiary education among women aged 25–34 in the early 1990s was low in Italy (6.6%), but significantly higher in Germany (19.5%), Poland (18.4%), and the United States (23.6%). An important interplay between education and employment career is represented by the percentage of young people not in education, employment, nor training (NEET). Among young women, NEET prevalence varied from 16.7% in the United States to 30.5% in Italy (see Table 1). Noteworthy, the proportion of inactive NEET (mainly comprising of NEETs due to family responsibilities and NEETs due to disability or illness) was more than half in all countries; in Italy, the share of long-term unemployed was substantial, too (Carcillo & Königs, 2015; Mascherini & Ledermaier, 2016).

In the 1980s and 1990s, the United States and Italy were two market economies characterised by a period of economic prosperity and the expansion of higher education. Work and family reconciliation was not supported by the state, but was either left to the market forces (as in the US case) or family (in Italy) (Korpi et al., 2013). East and West Germany constituted separate economic and political entities, only moving towards unification after the fall of the Berlin Wall in 1991. West Germany was classified as a conservative welfare state that supported a traditional male breadwinner model with part-time employment of women (Ostner, 1998). East Germany, like Poland, was under the communist rule until the end of the 1980s. Combining paid work and care in these settings was easier at that time due to low competition in the markets and due to widespread access to child care services (Leitner et al., 2008; Matysiak & Steinmetz, 2008). The fall of the communist regime resulted in an increase in work and family incompatibilities in both countries and substantial decline of fertility (Kotowska et al., 2008; Kreyenfeld, 2001).

## Data and Methods

For our analyses, we selected women aged 40 or over at the interview date who did not report having biological, adopted, or foster children at the moment of the interview. We took this decision so as to focus on women who have reached the age when childbearing is highly unlikely. Table 2 offers information about data sources and sample size. The four data sources were selected due to their comparability in terms of birth cohorts, and for the richness of information collected about respondent’s fertility, partnership, and employment histories. They are also large enough to ensure a non-negligible share of childless women.

**Table 2:** Data source and sample size information for Germany, Italy, Poland, and the United States

Country	Data source	Date of survey	Birth cohorts	Sample size
Germany	ALWA survey working and learning in a changing world	2007–2008	1956–1968	391
Italy	Household multipurpose survey on family and social subjects	2009	1956–1969	768
Poland	Gender and generation survey	2011	1956–1968	219
United States	National longitudinal survey of youth 1979	Multiple waves between 1979 and 2014	1957–1964	581

In order to describe the different life-course trajectories of childless women, we used sequence analysis (Abbott, 1995). We analysed education, employment, and partnership histories, and explored how these life spheres were intertwined by assigning a status to each month of each woman’s life between the ages of 15 and 40 with respect to these three spheres. Our state space, i.e. the set of all possible states that an individual could assume, consists of eight statuses constructed using the following characteristics:

- In education (yes/no), computed using the date when the highest degree was obtained^1^;
- Working (yes/no), computed using dates for each employment spell;
- In a co-resident union (yes/no), computed using dates for each union spell.

In the next step, we computed differences in life sequences between women using the dynamic Hamming distance (Lesnard, 2010). Similar to the commonly used optimal matching algorithm (Abbott & Tsay, 2000), the dynamic Hamming distance accounts for the duration of spells in the analysed sequences, but it is more sensitive to timing differences (i.e. union delay or union duration; Studer & Ritschard, 2016).

Once we reconstructed life-course sequences, we followed with discrepancy analysis (DA) (e.g. Struffolino et al., 2016; Studer et al., 2011), which allowed us to directly measure the life-course heterogeneity across countries. Using DA, we *quantify* the strength of the association between sequences and a time-invariant covariate (the country) by computing a pseudo-*R*^2^ value. Through 5000 permutation tests, we were able to ensure a precise measure of the strength of this association.

Findings from the DA then guided our analytical strategy to the final step of cluster analysis. If the pseudo-*R*^2^ value from DA were statistically significant, the cross-country heterogeneity in the life-course of childless women would be large enough to motivate a separate cluster analysis per country. If the DA results were not significant or null, a pooled sample of cluster analysis would be applied. In the last analytical step, we applied a cluster analysis to identify trajectories with similarities across the *timing*, *duration*, and *sequencing* of states among sequences. We employed Ward’s algorithm to create a universe of typical or ‘ideal-type’ life trajectories of childless women (Aassve et al., 2007). We used average silhouette widths to measure the coherence of the assignment of each sequence to a cluster (e.g. Devillanova et al., 2019; Raab & Struffolino, 2020).

We followed with sensitivity analyses to check the robustness of our findings. These were replicated using different distance metrics (i.e. optimal matching algorithm), and due to Poland’s smaller sample size, we repeated the analyses for Poland using an extended sample, including women born 1939–1971 (609 women in total). The results obtained from these additional analyses corroborate the findings reported in this manuscript (available upon request).

## Results

Our first step involved using a discrepancy analysis to quantify how much of the variance in the life-course sequences was accounted for by the country indicator. The estimated pseudo-*R*^2^ was equal to 0.04 (*p* value < 0.001 based on 5000 permutations), indicating that 4% of the variance in the computed sequences resulted from the country covariate. The country-effect was small but significant; therefore, for the cluster analysis, we grouped sequences for each country separately.

**Fig. 2:**
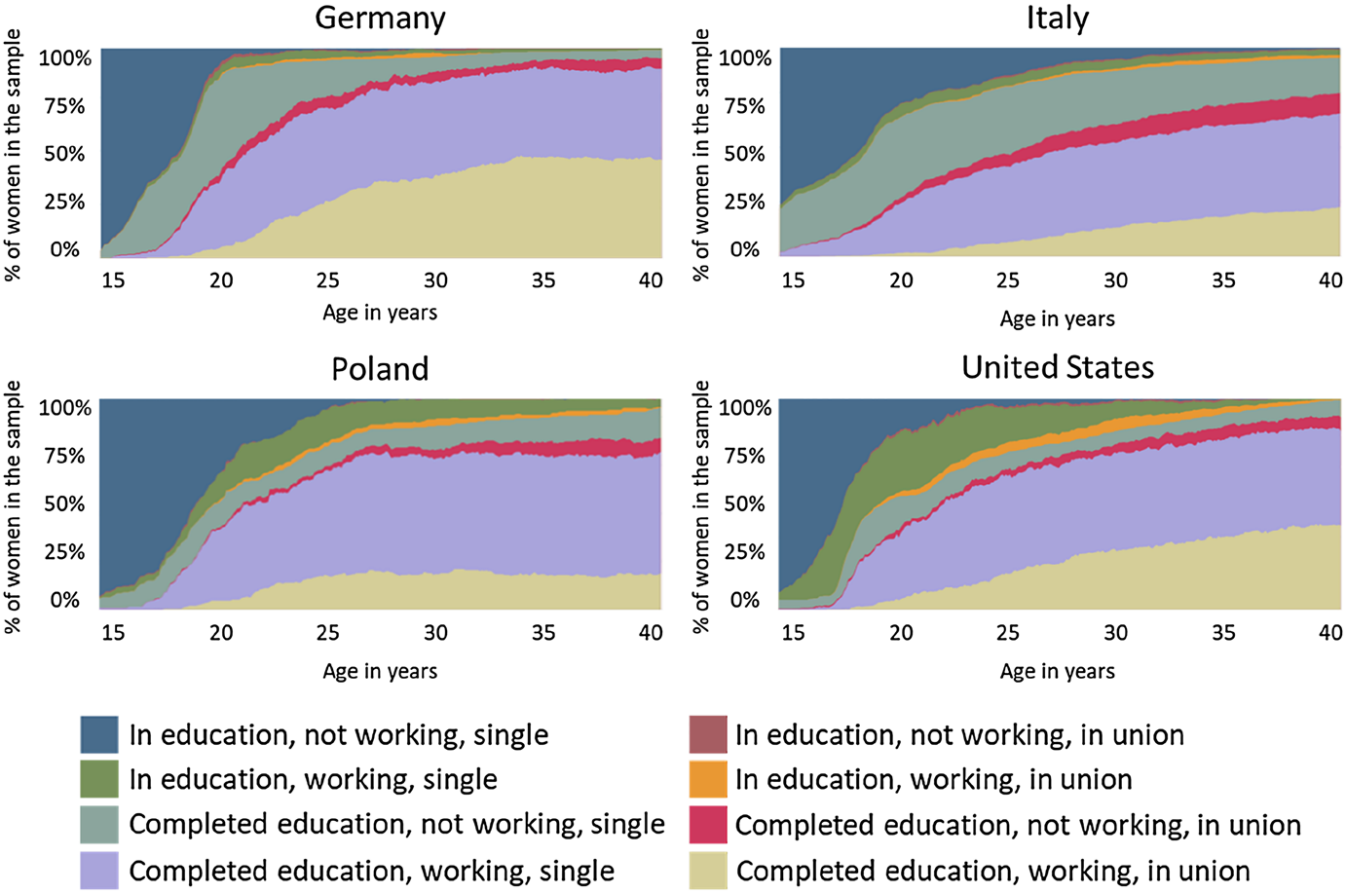
Chronograms representing life-course trajectories of childless women in Germany, Italy, Poland, and the United States. *Note:* The graph represents the distribution of the eight identified states within each country’s sample at each month from age 15 through 40

Figure 2 shows the life trajectories of childless women in each of the four countries. The descriptive differences in the life-courses of childless women across countries are visible as early as at age 15: while in Germany and Poland, the vast majority of women were still ‘in education, not working, single’, as much as 25% of women in Italy had already finished schooling. In the United States, already by age 15, we could distinguish a small proportion of women who worked without having concluded their education, but this group of women was not identified in any of the other countries. Many women in Poland and the United States completed their schooling later in life, presumably returning to school after a break.

Furthermore, women in the United States spent the most time working—on average 18.5 out of 25 years of observation—while women in Italy spent the least time working—on average 12.1 out of 25 years of observation. With respect to partnership history, Germany and the United States were characterised by marked spells of co-residential unions—in Germany, women spent approximately 8.4 years in a union, and 6.9 years in the United States. For comparison, women spent an average of only 4.6 years in co-residential unions in Italy and 4.4 years in Poland (see Table A1 in Online Appendix).

**Fig. 3:**
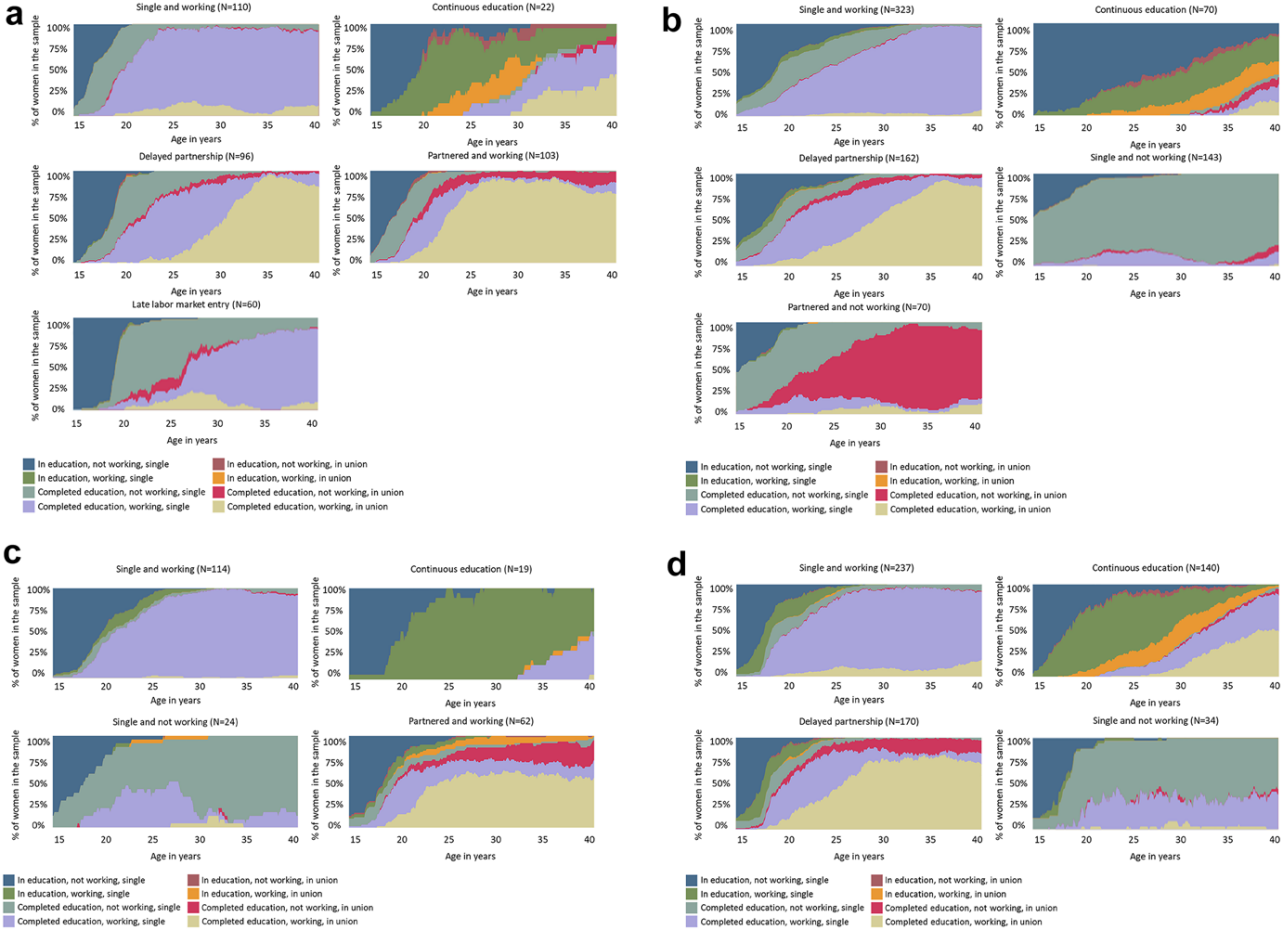
**a** Chronograms representing life-course trajectories of childless women across five clusters in Germany, **b** Chronograms representing life-course trajectories of childless women across five clusters in Italy, **c** Chronograms representing life-course trajectories of childless women across four clusters in Poland, and **d** Chronograms representing life-course trajectories of childless women across four clusters in the United States. *Note:* The graph represents the distribution of the eight identified states within each country’s sample at each month from age 15 through 40

Next, in order to identify the types of childless women’s life trajectories within each country, we proceeded with cluster analysis. Using average silhouette widths, we distinguished four distinct life-course trajectories of childless women in Poland and the United States, and five different childless women’s profiles in Germany and Italy (see Table 3 and Fig. 3; additional cluster characteristics are included in Online Appendix in Table A2-A5). In total, seven life-course trajectories were identified. Interestingly, some clusters across the countries shared similarities and we grouped them into one category. Other clusters emerged as unique to a specific country context. We follow with a detailed description of the identified clusters.

**Table 3:** Life-course profiles of childless women. Percentage values and total absolute values. Germany, Italy, Poland, and the United States

Universal cluster	Germany	Italy	Poland	United States
*Single and working* (They left the educational system before the age of 20, and spent most of their adult life working and without a partner.)	28.1	42.1	52.1	40.8
*Continuous education* (They combined education and work, spending most of their twenties both working and in education. Their union status varied over the life-course.)	5.6	9.1	8.7	24.1
Total (*n*)	33.8 *(132)*	51.2 *(393)*	60.7 *(133)*	64.9 *(377)*

Country-specific cluster	Germany	Italy	Poland	United States
*Delayed partnership* (They worked and stayed in education up to the ages of 22–24. They partnered later in their adult life.)	24.6	21.1		29.3
*Single and not working* (They left the educational system before the age of 20, did not enter into a union, and did not work.)		18.6	11.0	5.9
*Partnered and working* (They partnered over the life-course and continuously worked. They varied in terms of time spent in education.)	26.3		28.3	
*Late labour market entry* (They spent longer time in education, up to the ages of 22–24, and experienced a non-employment spell between the end of education and labour market entry, which happened late. They were not in a union.)	15.3			
*Partnered and not working* (They spent most of their life-course in unions, and finished education between the ages of 18 and 20. They did not work over the life-course.)		9.1		
Total (*n*)	66.2 *(259)*	48.8 *(375)*	39.3 *(86)*	35.1 *(204)*

First, we considered the two clusters present in all four countries. The first category, *Single and working,* was the most numerous cluster in each country, comprising 28.1% of German women to 52.1% of Polish women. Women belonging to this cluster completed their education at a relatively young age (compared to their country peers), and spent most of their adult life working and without a co-resident union.

The second universal cluster, *Continuous education*, was especially prevalent in the United States, where 24.1% of childless women were assigned to it. In comparison, fewer than 10% of childless women were assigned to this group in the three remaining countries. This cluster consisted of women who, compared to the other childless women in the respective country, either spent a longer period in education in young adulthood or returned to education later in life. Interestingly, many women in this cluster in Italy spent a considerable amount of their adult lives outside of employment. Union status in this cluster also varied over the life-course and among countries: while in Germany, Italy, and the United States it comprised both single women and those in co-residing unions, in Poland nearly all women did not enter into a union.

The remaining clusters were not detected in all four countries. The *Delayed partnership* cluster was identified in Germany, Italy, and the United States. Women in this cluster significantly postponed union entry compared to their country peers. In each of these three countries, it consisted of a relevant proportion of childless women—from 21.1% in Italy to 29.3% in the United States. Noteworthy, while the patterns of behaviour were similar in the three countries, the postponement was much less pronounced in the United States than in the two other countries. In all three countries, childless women in this group experienced a marked period of non-working in their 20 s.

The *Single and not working* profile was characterised by women who completed their education at a young age, and remained both single and outside of the labour market. We identified the cluster in Italy, Poland, and the United States, although it was residual in the latter country (5.9% compared to 11.0% in Poland and 18.6% in Italy). Women belonging to this cluster shared a life-course pattern highly similar to the NEET youth for both educational and employment careers.

We identified the *Partnered and working* cluster that accounted for a large proportion of childless women both in Germany and Poland (26.3% and 28.3%, respectively). It described a traditional pattern in the transition to adulthood, involving women who had completed their education, began working, and entered unions at a young age (compared to their country peers). Moreover, we identified a unique *Late labour market entry* cluster in Germany. This group consisted of 15.3% of German childless women. While they studied for longer, they faced difficulties upon entry into the labour market, living a prolonged period of non-employment before starting work during their late 20s, while not entering into a union. Finally, we detected a small cluster (9.1%) of *Partnered and not working* women in Italy. This group was formed by women who completed their education at a young age, did not work, and entered a union relatively early (during their 20s).

## Discussion

In this paper, we concentrated on four distinct country settings: Germany, Italy, Poland, and the United States, to examine the life-course and cross-country heterogeneity in the lives of childless women. In line with previous research, across all countries we found childlessness to be strongly linked to singlehood, but also characterised by a complex set of intersections of relationship, employment, and education histories across the life-course. Specifically, in each country, we identified four or five distinct life-course patterns among childless women. Consequently, the childless universe does not appear to be uniquely formed by highly educated, working women. In all countries, single women with low or medium educational attainment and single women with weak attachment to the labour market made up a marked proportion of the childless universe.

The revealed diversity in a childless life-course can have important implications for women. While some childless women might accumulate resources over their life-course, others might instead deplete them, for example, by coping with lack of a partner or employment. Such differences in accumulated capital might translate into tangible variation in the economic and personal well-being throughout adulthood and into old age. For instance, being childless could imply faded intergenerational supports in old age, which could cause severe problems in a society where the welfare is largely based on these networks (such as Italy and Poland).

The differences between childless women in the analysed countries potentially reflected general discrepancies in terms of education and labour market systems. For instance, the larger proportion of single professional childless women identified in the United States may relate to tertiary education having been more prevalent there—for men and women, as well as mothers and the childless. The presence of a unique cluster of childless women with a delayed labour market entry in Germany might reflect particular labour market conditions in this setting, during the period of economic change following the unification.

However, some of the observed cross-country differences showed a more nuanced picture. For example, while first-time marriage rates were comparable across the four countries at the time when the women analysed reached adulthood, we observed a larger proportion of partnered childless women in Germany and the United States. This finding can signal a looser link between marriage and parenthood in these countries, compared to Italy and Poland, potentially linked to institutional differences such as broader access to hormonal contraception and sterilisation, and/or cultural variation around individual preferences for partnership and childlessness.

Importantly, while we found country-level differences in the childless life-course, we also observed descriptive similarities across the countries. In addition to a group of single and working women present in all four settings, a group of women who prolonged their education (compared to their country peers) was also identified across all countries. Put together, these findings provide descriptive evidence for both *country-specificity* and *cross-country similarity* in the pathways to childlessness.

Our study has several limitations. While we analysed four countries across different institutional, cultural, and geographical settings, we did not include a Northern European country, which could have provided valuable information on the universality of some of the pathways to childlessness. Northern European countries are characterised by distinct social welfare programmes, and fertility rates markedly different from those in the four analysed countries (Billingsley & Ferrarini, 2014; Thévenon & Gauthier, 2011). Nevertheless, childlessness in Nordic countries has received scholarly interest (Jalovaara & Fasang, 2017; Neyer et al., 2017; Rotkirch & Miettinen, 2017).

Furthermore, our measure of education could not account for intermittent schooling spells or returns to education later in life, instead classifying women as being continuously in education. We also did not distinguish between part-time and full-time employment, or marital and non-marital unions—which could be included in future studies. Consistent measures of heterosexual living-apart-together relationships or same-sex unions (both co-residing and non-resident) were also missing in the data sources which we used.

Additionally, due to data limitations, we could not investigate neither women’s reproductive health (as well as that of the partner), nor the degree of agreement/disagreement in reproductive plans between partners. Given our analyses, we could speculate that a share of women involved in the trajectory of not working, single women could have significant health problems; their illness could affect both their likelihood of being partnered and of being on the labour market (Mynarska et al., 2015). Analogously, a share of partnered, not working women could have relevant family care burdens towards parents or other relatives that impede work-related commitments or other family-related commitments, such as parenthood. Finally, some couples could have encountered potential reproductive health problems—all speculations that should be investigated with proper information. However, being childless does not exclude childrearing during a woman’s life-course, especially if partnered.

The limitations of our study highlight important challenges for future research in terms of the more detailed and comparative data collection about the childless life-course. Regardless of these limitations, this study has demonstrated the potential and benefits of analysing a process of remaining childless in a comparative perspective, while not neglecting within-country heterogeneity of childless women’s biographies. The identification of different types of childless women across space, and—possibly—over time, can generate important insights about life-course developments leading to childlessness.

## Supplementary Information

Below is the link to the electronic supplementary material.

Supplementary file1 (DOCX 440 kb)

## Footnotes

1 Consequently, women who left education early but then returned in later life were classified as being continuously in education until they finalised their highest degrees. This is an important shortcoming of this data set as it demonstrates the complexity of harmonising life-course trajectories across country-specific data sources. Because education has been identified as an important component of childless life, we have decided to nonetheless include this measure in our analyses, along with careful discussions of its implications throughout our manuscript.
