# Supplementary material for: Life-Course Trajectories of Childless Women: Country-Specific or Universal?
Source: Eur J Popul. 2022 Jun 9;38(5):1315–32. doi: 10.1007/s10680-022-09624-5 (PMC9726798; doi:10.1007/s10680-022-09624-5)
Supplement: Supplementary file 1 — Supplementary file1 (DOCX 440 kb) [file 10680_2022_9624_MOESM1_ESM.docx]

**Online Appendix**

**Appendix A: Descriptive statistics of sequences and clusters**

**Table A1:** Mean time spent in education, working, and in co-residential unions between ages 15 and 40. Childless women in Germany, Italy, Poland, and the United States.

|  | Germany |  | Italy | Poland | US |
| --- | --- | --- | --- | --- | --- |
| Mean number of years spent until the highest educational level is completed | 4.0 |  | 5.8 | 7.5 | 7.6 |
| Mean number of years spent working | 16.8 |  | 12.1 | 16.6 | 18.5 |
| Mean number of years spent in a co-residential union | 8.4 |  | 4.6 | 4.4 | 6.9 |

**Table A2:** Sequence analysis descriptive measures of identified clusters. Germany.

|  | Mean number of years spent until the highest educational level is completed | Mean number of years spent working | Mean number of years spent in a co-residential union |
| --- | --- | --- | --- |
| Single and working | 2.4 | 18.8 | 1.9 |
| Continuous education | 17.2 | 17.8 | 6.7 |
| Delayed partnership | 3.7 | 16.7 | 10.4 |
| Single and not working | N/A | N/A | N/A |
| Partnered and working | 2.7 | 17.8 | 17.3 |
| Late labour market entry | 4.7 | 11.5 | 6.7 |
| Partnered and not working | N/A | N/A | N/A |

Note: This is between ages 15 and 40. If a cell is blank, that cluster has not been identified for that country.

**Table A3:** Sequence analysis descriptive measures of identified clusters. Italy.

|  | Mean number of years spent until the highest educational level is completed | Mean number of years spent working | Mean number of years spent in a co-residential union |
| --- | --- | --- | --- |
| Single and working | 5.5 | 16.0 | 0.7 |
| Continuous education | 22.9 | 9.6 | 4.2 |
| Delayed partnership | 4.1 | 18.1 | 11.8 |
| Single and not working | 2.1 | 2.1 | 0.6 |
| Partnered and working | N/A | N/A | N/A |
| Late labour market entry | N/A | N/A | N/A |
| Partnered and not working | 1.9 | 3.2 | 14.4 |

Note: This is between ages 15 and 40. If a cell is blank, that cluster has not been identified for that country.

**Table A4:** Sequence analysis descriptive measures of identified clusters. Poland.

|  | Mean number of years spent until the highest educational level is completed | Mean number of years spent working | Mean number of years spent in a co-residential union |
| --- | --- | --- | --- |
| Single and working | 6.5 | 18.5 | 0.3 |
| Continuous education | 23.0 | 18.9 | 0.2 |
| Delayed partnership | N/A | N/A | N/A |
| Single and not working | 3.9 | 5.6 | 0.8 |
| Partnered and working | 6.0 | 16.6 | 14.8 |
| Late labour market entry | N/A | N/A | N/A |
| Partnered and not working | N/A | N/A | N/A |

Note: This is between ages 15 and 40. If a cell is blank, that cluster has not been identified for that country.

**Table A5:** Sequence analysis descriptive measures of identified clusters. The United States.

|  | Mean number of years spent until the highest educational level is completed | Mean number of years spent working | Mean number of years spent in a co-residential union |
| --- | --- | --- | --- |
| Single and working | 5.2 | 19.8 | 1.9 |
| Continuous education | 16.8 | 19.4 | 7.3 |
| Delayed partnership | 4.1 | 18.3 | 14.9 |
| Single and not working | 3.7 | 7.4 | 0.8 |
| Partnered and working | N/A | N/A | N/A |
| Late labour market entry | N/A | N/A | N/A |
| Partnered and not working | N/A | N/A | N/A |

Note: This is between ages 15 and 40. If a cell is blank, that cluster has not been identified for that country.

**Appendix B: Cluster analysis for the pooled sample across four countries**

In this Appendix, we present results for cluster analysis for the pooled sample of childless women across the four countries: Germany, Italy, Poland, and the United States. We use data and methodologies described in the main body of the manuscript. Through a comparison of several cluster solutions for the pooled sample using silhouette widths, we chose the five cluster solution.

**Figure B1**: Chronogram representing life-course trajectories of childless women in the pooled sample.


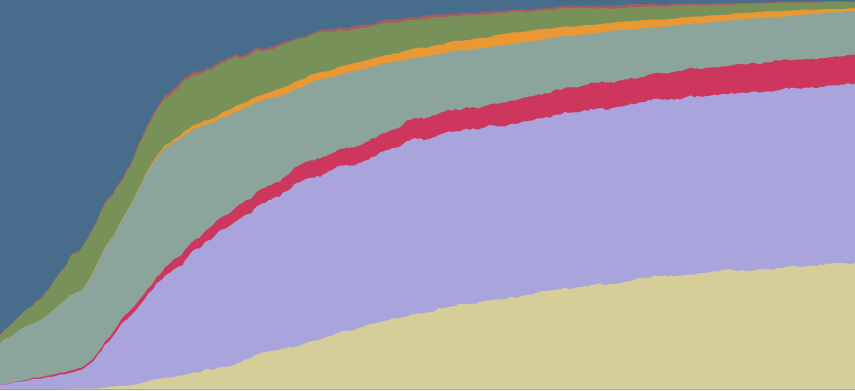


% of women in the sample

100%

75%

50%

25%

0%


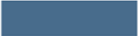


In education, not working, single

In education, working, single


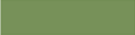

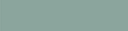

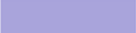


Completed education, not working, single

Completed education, working, single

In education, not working, in union

In education, working, in union

Completed education, not working, in union

Completed education, working, in union


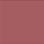

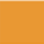

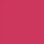

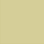


15

20

25

30

35

40

Age in years

Pooled sample (N=1,959)

*Note: The graph represents the distribution of the eight identified states within the pooled sample at each month from age 15 through 40.*

**Figure B2:** Chronograms representing life-course trajectories of childless women across 5 clusters in the pooled sample


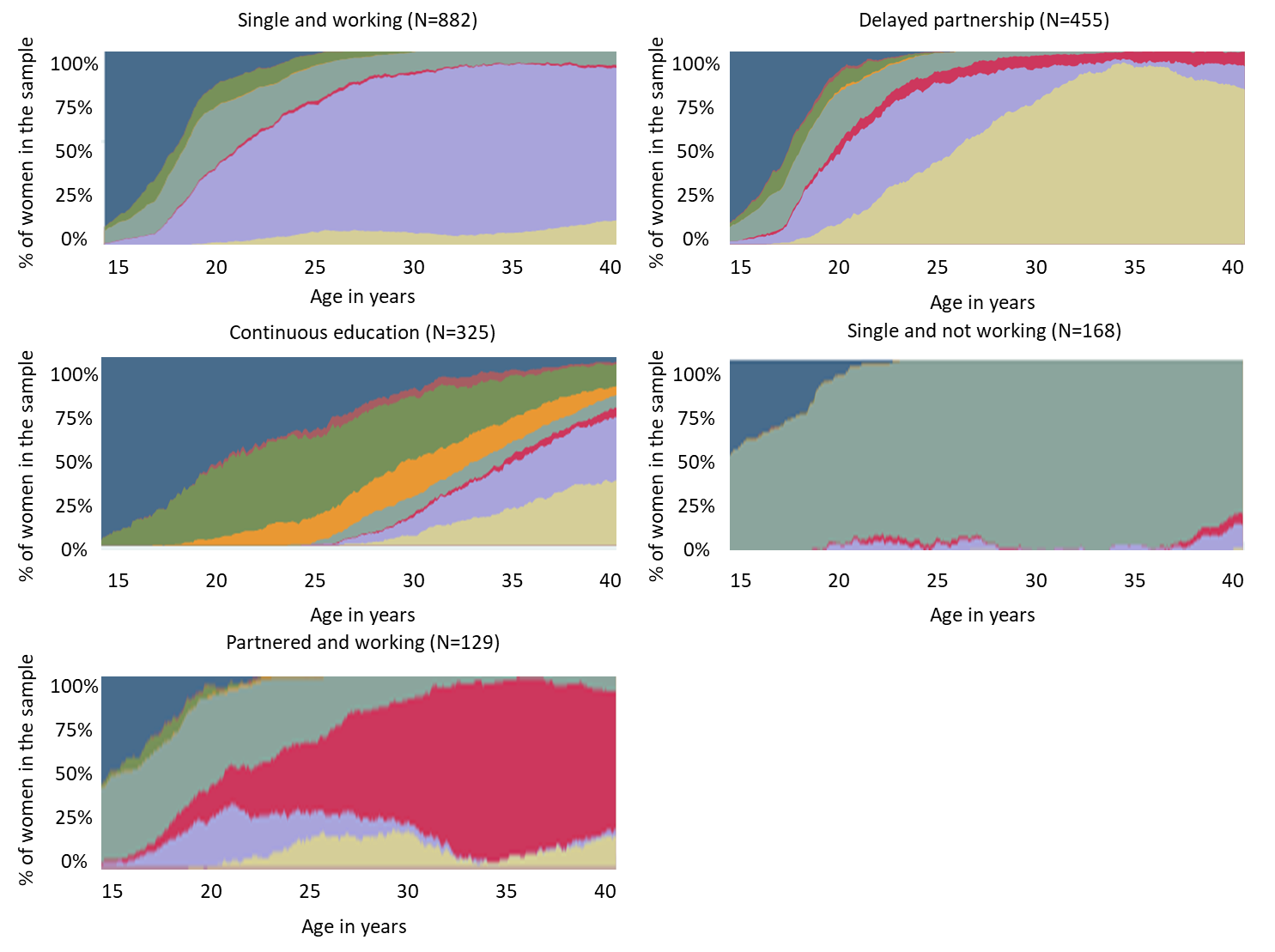


*
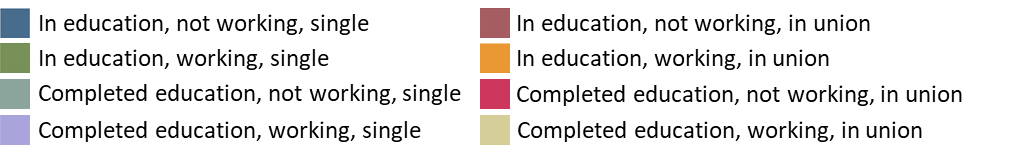
*

*Note: The graph represents the distribution of the eight identified states within the pooled sample at each month from age 15 through 40.*

**Table B1**: Life-course profiles of childless women in the pooled sample from cluster analysis.

| Cluster | Pooled sample |
| --- | --- |
| ***Single and working***  (They left the educational system before the age of 20, and spent most of their adult life working and without a partner.) | 45.0% |
| ***Delayed partnership***  (They worked and stayed in education up to the ages of 22–24. They partnered later in their adult life.) | 23.2% |
| ***Continuous education***  (They combined education and work, spending most of their twenties both working and in education. Their union status varied over the life-course.) | 16.6% |
| ***Single and not working***  (They left educational system before the age of 20, did not enter into a union, and did not work.) | 8.6% |
| ***Partnered and working***  (They partnered over the life-course and continuously worked. They varied in terms of time spent in education.) | 6.6% |
| **Total**  ***(n)*** | ***100.%***  ***(1,959)*** |
